# The effect of tail stiffness on a sprawling quadruped locomotion

**DOI:** 10.3389/frobt.2023.1198749

**Published:** 2023-08-24

**Authors:** Josh Buckley, Nnamdi Chikere, Yasemin Ozkan-Aydin

**Affiliations:** ^1^ Department of Biomedical Engineering, University of Galway, County Galway, Ireland; ^2^ Department of Electrical Engineering, University of Notre Dame, Notre Dame, IN, United States

**Keywords:** bio-inspired robotics, quadruped locomotion, flexible tail, variable stiffness, stability, sprawled posture

## Abstract

A distinctive feature of quadrupeds that is integral to their locomotion is the tail. Tails serve many purposes in biological systems, including propulsion, counterbalance, and stabilization while walking, running, climbing, or jumping. Similarly, tails in legged robots may augment the stability and maneuverability of legged robots by providing an additional point of contact with the ground. However, in the field of terrestrial bio-inspired legged robotics, the tail is often ignored because of the difficulties in design and control. In this study, we test the hypothesis that a variable stiffness robotic tail can improve the performance of a sprawling quadruped robot by enhancing its stability and maneuverability in various environments. In order to validate our hypothesis, we integrated a cable-driven, flexible tail with multiple segments into the underactuated sprawling quadruped robot, where a single servo motor working alongside a reel and cable mechanism regulates the tail’s stiffness. Our results demonstrated that by controlling the stiffness of the tail, the stability of locomotion on rough terrain and the climbing ability of the robot are improved compared to the movement with a rigid tail and no tail. Our findings highlight that constant ground support provided by the flexible tail is key to maintaining stable locomotion. This ensured a predictable gait cycle, eliminating unexpected turning and slipping, resulting in an increase in locomotion speed and efficiency. Additionally, we observed the robot’s enhanced climbing ability on surfaces inclined up to 20°. The flexibility of the tail enabled the robot to overcome obstacles without external sensing, exhibiting significant adaptability across various terrains.

## 1 Introduction

Tails have been observed to serve a multitude of functions in various extinct and extant animal species, ranging from mating and defense to aiding in locomotion (Hickman, 1979; Santori et al., 2005; Libby et al., 2012; McInroe et al., 2016; Ibrahim et al., 2020). Some animals, such as lizards, can detach their tails as a defensive mechanism to distract predators (Arnold, 1984; Naya et al., 2007; Higham et al., 2013). Tails also aid in maintaining balance (Hickman, 1979; Walker et al., 1998) and stability (JesseYoung et al., 2021), especially during climbing or navigating through dense vegetation, and provide maneuvering (Gillis and Higham, 2016) and grasping (Garber PaulA. and Rehg JenniferA., 1999) abilities. For example, many species of monkeys use their prehensile tail to grasp branches to support themselves while feeding (Garber P. A. and Rehg J. A., 1999; Schmitt et al., 2005).

In many cases, tails serve as a crucial propulsion system in both aquatic (Lighthill, 1969; Lauder and Tytell, 2005; Flammang et al., 2011; Feilich and GeorgeLauder, 2015) and terrestrial environments (Hickman, 1979; Santori et al., 2005; McInroe et al., 2016; Ibrahim et al., 2020). Fish use their tails to move through the water by undulating antagonistic muscles that control tail oscillation (Lauder and Tytell, 2005). Additionally, fish use their tails for steering and maintaining balance, making quick movements and turning to avoid obstacles or predators (Keenleyside, 2012). Similarly, the tail assists in locomotion activities of terrestrial animals such as quadrupedal walking (Willey and Blob, 2004; Karakasiliotis and Jan Ijspeert, 2009; Higham et al., 2013), climbing (Chang-Siu et al., 2011), jumping (Alfred Brazier Howell, 1944; Gillis et al., 2013), aerial descent (Young et al., 2015), and gliding (Siddall et al., 2021). Some legged animals, such as kangaroos, utilize their tails for balance during bipedal hopping. However, when they move at a slower pace, like when grazing or interacting with others, they use their tails as a “fifth leg.” The tail gives them an extra point of contact with the ground and an additional propulsive force (Karakasiliotis and Jan Ijspeert, 2009; O’Connor et al., 2014).

**FIGURE 1:**
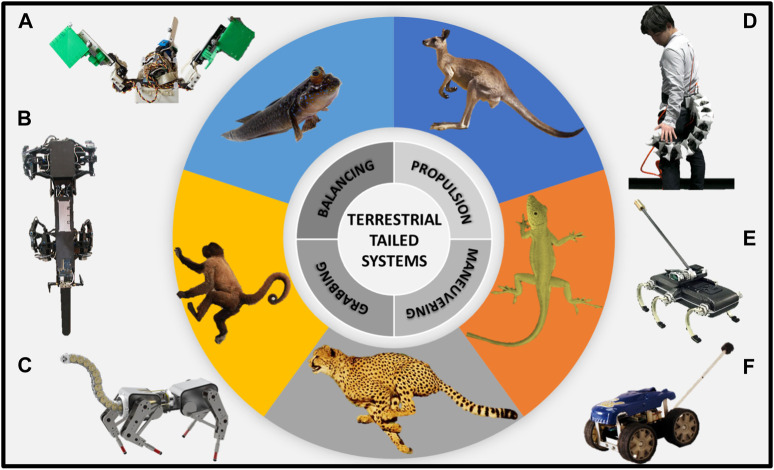
Examples of terrestrial-tailed animals and robots. The robotic systems depicted in the figure were inspired by the anatomical and behavioral characteristics of the animals featured in the central image. **(A)** Muddybot inspired by mudskipper fish to demonstrate the function of a tail on the locomotion of granular surfaces (McInroe et al., 2016), **(B)** RISE Ver.3 climbing robot (Haynes et al., 2009), **(C)** Articulated-tailed quadruped robot (Ben-Tzvi and Liu, 2021), **(D)** Prosthetic tail to extend human body functions (Nabeshima and MHD Yamen Saraiji Minamizawa, 2019), **(E)** Tailed hexapod robot (Johnson et al., 2012), **(F)** Tailbot, a lizard-inspired tailed quadruped robot (Chang-Siu et al., 2011).

These natural tail functionalities have served as a source of insight and inspiration for the development of novel robotic systems (Figure 1) that emulate and replicate the natural movements and functionalities of the biological organisms (Nabeshima and MHD Yamen Saraiji Minamizawa, 2019; Ben-Tzvi and Liu, 2021). The research conducted thus far has primarily centered around tail designs aimed at enabling inertial adjustments to enhance balance and stability (Briggs et al., 2012; Joseph et al., 2021), or maneuverability when turning (Jusufi et al., 2010; Johnson et al., 2012; Casarez et al., 2013; Patel and Braae, 2013; Patel and Braae, 2013; Casarez and Fearing, 2017), running (Chang-Siu et al., 2011), jumping (Brill et al., 2015) and climbing (Haynes et al., 2009; Lynch et al., 2012). There has been limited research with respect to tails providing stability via continuous ground support and tails that directly aid in propulsion. In (Soto et al., 2022), a tail was designed that periodically “taps” off the ground, providing a momentary ground contact. This design provided some success in obstacle navigation and releasing the robot when trapped.

Although there are several potential advantages of using tail-like appendages on robots, challenges need to be overcome to realize the potential of tailed systems fully (Liu and Ben-Tzvi, 2021; Schwaner et al., 2021). For example, one of the challenges in developing bio-inspired robotic tails is designing them to be flexible and agile enough to provide the desired benefits, without adding too much weight or complexity to the overall system. In addition, we need to consider how to integrate the tail into the overall control system of the robot and how to power it. There is also a need for more sophisticated control algorithms and sensors that can accurately sense and respond to the movements and forces acting on the tail during locomotion.

In this paper, we study how the flexibility of the tail can enhance the locomotion performance of a sprawled quadruped robot used in (Ozkan-Aydin and Goldman, 2021) in various environments. We developed a 3D-printed, underactuated articulated tail, capable of modulating stiffness using a servo-actuated cable-driven mechanism. Our tail design was inspired by the curved tail of kangaroos, which comprises several caudal vertebrae and provides excellent control when hopping and walking and physical support when grazing (O’Connor et al., 2014). Throughout the prototyping stage of our robot’s tail, we observed that an excessive number of vertebra-like segments resulted in an overabundance of flexibility. This observation aligns with the findings regarding dinosaurs’ tails reported in (Hone et al., 2021). Subsequently, the reduced number of such segments is sufficient for emulating the characteristic behavior of a biological vertebrate tail. It is important to note that our robot embodies a deliberate simplification of an underactuated quadrupedal robot design. Owing to the intersegmental coupling of the legs, only a singular leg within each segment maintains contact with the ground during locomotion. Therefore, the robot inherently manifests an unstable nature, as just two opposite legs engage with the ground at any given moment.

While numerous quadruped robots heavily rely on computationally demanding procedures and intricate gait algorithms (Raibert, 1990; Rebula et al., 2007; Raibert et al., 2008; Alexander et al., 2013; Focchi et al., 2017; Carlo et al., 2018; Winkler et al., 2018; Chai et al., 2022), our robot employs a straightforward open-loop diagonal gait (diagonal legs on the ground at each time step). By integrating an underactuated flexible tail, we aim to enhance the robot’s performance without necessitating the utilization of complex gait algorithms, an abundance of sensory input, or additional actuators. Incorporating a flexible tail holds significant potential for improving the locomotion capabilities of diverse quadrupedal robot configurations. Specifically, by accommodating variations and disturbances encountered during locomotion, the flexible tail empowers the robot to adapt and maintain stability, ensuring a more seamless traversal through complex terrains. The flexibility of the tail increases the robustness. It enables dynamic responses to external forces, augmenting the robot’s ability to navigate uneven surfaces, inclined and obstacle environments, or unpredictable natural terrains. Thus, incorporating a flexible tail could present a substantial opportunity to enhance the overall locomotion capabilities and adaptability of quadrupedal robots operating within challenging environments. This is particularly significant in scenarios where achieving stable navigation poses inherent difficulties or when using a predefined stable gait tailored for smooth surfaces proves inadequate.

## 2 Materials and methods

### 2.1 Body and leg design

**FIGURE 2:**
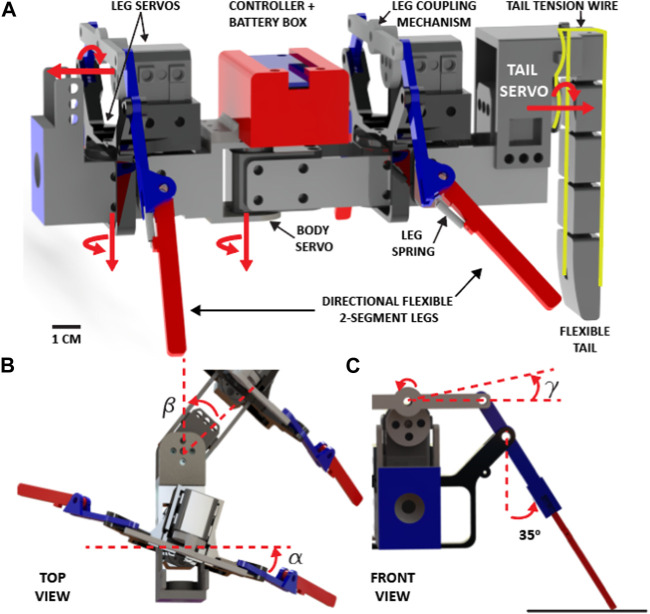
A sprawling quadruped robot combined with a multi-segment flexible tail. **(A)** A computer-aided design of the fully autonomous quadruped robot (about 20 cm long) that includes two body segments with a pair of directional flexible legs connected via an XL-320 servo motor. A 7.4 V LiPo battery and Robotis Open-CM 9.04 controller are placed into the box on the top of the body servo (red box). Two XL-320 servos control the horizontal and vertical motions of the coupled legs in a segment. A flexible, multi-segmented, cable-driven tail is attached to the back of the robot. Red arrows show the rotation axis of the leg and body servos (Supplementary Video/0:03-0:07 s), **(B)** the top view of the front segment of the robot with leg swing (for/aft) angle (*α*) and body angle (*β*), **(C)** the front view of the leg when it is in a neutral position (red arrows show the rotation axis), (*γ*) is the leg up/down angle.

In this study, we added a multisegmented tail to the quadruped robot used in the previous studies (Ozkan-Aydin et al., 2020; Ozkan-Aydin and Goldman, 2021). The robot has two body segments, each consisting of two directional flexible multi-jointed legs (total length = 12 cm) that are coupled via a rigid 1-DoF mechanism (Figures 2A, B). Springs (spring constant = 0.2 kg/cm, McMaster; product number, 9654K949) that are connected between the upper and lower part of the leg joints allow the legs to bend when encountering obstacles and then return to a neutral position passively once the obstacle has been traversed (Ozkan-Aydin et al., 2020). When the legs are at their neutral angle (i.e., the leg coupling mechanism is parallel to the ground, Figure 2C), the height of the center of mass (CoM) of the robot is 5 cm. All of the robot parts were 3D-printed with a Stratasys F170 printer using ABS material.

The robot consists of a Robotis OpenCM 9.04 microcontroller, a lithium polymer battery (11.1 V, 1,000 mAh), six Robotis Dynamixel XL-320 motors (stall torque is 0.39 N m): one for controlling the rotation of the body, one for controlling tail stiffness, and four for controlling the vertical and horizontal motion of the legs at the front and back segments Figure 2A. The leg pair in each segment is coupled via rigid links, as shown in Figure 2A. This coupling entails coordinating vertical and horizontal motion by utilizing shared servo motors. When the left leg is in the air phase, the right leg remains on the ground, and *vice versa*. The same principle applies to horizontal motion, where the backward movement of one leg accompanies the forward movement of the other leg.

Because of the mechanical constraints, the rotational range of the legs in the horizontal plane is constrained to a maximum of *α* = −30^*o*^ to 30^*o*^, while the body servo possesses the ability to rotate within the range of *β* = −30^*o*^ to 30^*o*^ from their respective neutral positions, as illustrated in Figure 2B. The servos that control the vertical motion of the legs can rotate *γ*_max_ = 30^*o*^ (Figure 2C), which raises the tip of the legs about 4 cm above the ground.

### 2.2 Rigid and flexible tail designs

In our experiments, we aimed to elucidate the advantages and potential benefits of the flexible tail compared to its rigid counterpart. As part of this investigation, we developed a rigid, stick-like tail that has a similar structure to the legs. This design choice allowed for a controlled assessment of the flexible tail’s performance and its impact on locomotor capabilities.

Within the rigid tail design, the tail is affixed to the tail servo, thereby enabling control over the left-right motion of the tail. In contrast, within the flexible tail design, the same servo is utilized to modulate the stiffness of the flexible tail rather than controlling its lateral movement.

#### 2.2.1 Rigid tail

**FIGURE 3:**
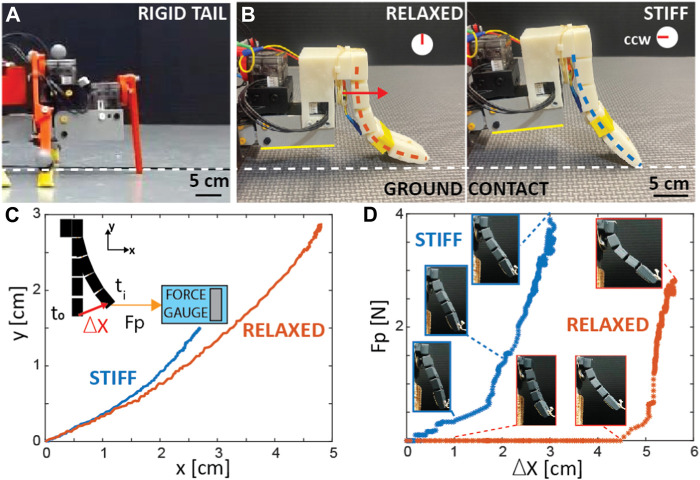
Rigid and Variable Flexible Tail. **(A)** Rigid tail **(B)**. States of the flexible tail. Tail stiffness increases from left to right by turning the tail servo in a counter-clockwise (CCW) direction along the axis shown with a red arrow, causing the height of the rear of the body to increase (Supplementary Video/0:08-0:52 s). **(C)** Horizontal (x) vs. vertical (y) displacement of the tip of the tail when it is stiff (blue) and relaxed (red) with respect to its initial position. The inset figure illustrates the setup for measuring the pulling force (*F*_*p*_). **(D)** Pulling force (*F*_*p*_) vs. absolute distance of the tip with respect to its initial position. Insets show the shape of the tail after pulling (Supplementary Video/0:35-0:52 s).

The rigid tail was designed to be of similar shape to the robot’s legs (rectangular) but greater in length (90 mm in length, 3.5 mm thick, and 8 mm wide, Figure 3A). The top of the tail was connected to the rear servo motor. The bottom of the tail was filleted, with the removal of sharp corners key to ensuring the tail does not get stuck on obstacles as it moves. The primary goal of this design was to provide an extra contact point at the back section of the robot. Since the legs are coupled, only one of the legs of each segment is on the ground at each time step (Ozkan-Aydin and Goldman, 2021), and therefore a support triangle cannot be formed. Without a tail, the diagonal gait of the robot was interrupted and led to instability during locomotion (Ozkan-Aydin and Goldman, 2021), with the body of the tail striking the ground twice per gait cycle. This also led to foot slippage and insufficient ground clearance, which leads to problems when the robot attempts to climb and navigate obstacles.

The rigid tail has a passive role throughout the smooth surface experiments, serving as an additional contact point along the robot’s back. However, it is noteworthy that the lateral motion of the rigid tail (undulation of the tail from left to right like a lizard’s tail) could be actively controlled through the tail servo motor. This mechanism could be useful for climbing or walking on rough terrain. If the robot gets stuck on an obstacle, it can be pushed out of that position by repeatedly rotating its rigid tail from side to side.

#### 2.2.2 Flexible tail—mechanism design and actuation

The tail comprises five articulating vertebra-like segments of cuboidal shape (Figure 2). The fifth segment of the tail has a narrow, curved end, which is in contact with the ground. This segment includes a fillet on the interior face, allowing the tail to pass over obstacles and sharp ledges without causing the robot to get stuck. The tail was 3D printed using a Stratasys F170 printer with all parts intact. The links between each segment were designed to eliminate the need for assembly. Dimensions of the linkage component can be modified in order to alter the rigidity of the tail along with the range of motion.

Included in the design is a connection component, a box attached to the back of the robot. This component allowed the tail to attach and detach from the robot with ease. Furthermore, it elevates the attachment point of the tail significantly above the body. This is important as it allows a longer tail to be developed, leading to an increased range of motion and, thus, more effective robot locomotion. The connection component also includes small loops at the top and the side, which helped guide the cabling when wrapping around the reel.

To control the flexibility of the tail, we designed a mechanism that consists of a custom-made reel that is attached to the rear servo motor (Figures 2, 3B, Supplementary Video/0:08-0:52 s). Kevlar wire was then threaded through the front and rear channels of the tail and wrapped around the reel attached to the servo motor. When the servo motor was in its initial position, the tail was relaxed and very flexible (Figure 3B-left). The wires were wrapped around the reel in the same direction (counterclockwise (CCW)). The tension in the cables was carefully tuned so that when the reel rotated CCW 90°, the tail would be pulled into an approximate “s” shape (Figure 3B-right), similar to the shape of vertebrate tails observed in certain biological species such as the kangaroo (O’Connor et al., 2014). With an applied rotation of the tail servo motor of between 0 and 90° (CCW), the distance between the segments was reduced, increasing the rigidity (or decreasing the tail’s flexibility).

We conducted measurements of the horizontal pulling force against the displacement of the tip of the tail using a force gauge (see Figure 3C). As seen from Figures 3C, D, the relaxed tail can bend about 45° from its initial position with about zero pulling force (Supplementary Video). Furthermore, it is observed that the stiff tail configuration can exert approximately 1 N of normal force, while the relaxed tail configuration can only apply a significantly reduced force of 0.1 N (Supplementary Video/0:08-0:52 s). Two different implementations of this approach were tested, primarily the effect of periodically stiffening and relaxing the tail during key phases of the gait cycle and the effect of using a touch sensor to sense when the tail should stiffen and relax. More detail on the stiffening/relaxation timing can be found in Sections 3.3.3 and Section 3.3.4.

#### 2.2.3 Flexible tail—tail states and actuation patterns

Due to the tail’s flexibility, the rigidity could be carefully controlled and altered via the actuation of the servo motor. Three unique tail states were used across the experiments: fully relaxed (or flexible), high rigidity, and periodic stiffening and relaxation.

The first two states were introduced at the end of Section 2.2.2 and in Figures 3B–D. The fully relaxed state involves no servo motor rotation and keeps it at its default position. As a result, there was no tension in the cables, allowing the tail to act passively and remain flexible. The high rigidity state was the inverse of the fully relaxed state, with the servo motor maintained at a constant rotation of 90° anticlockwise from its original position. The desired rigidity and tail shape can be achieved by choosing the appropriate degree of rotation within this a 0 to 90 degree range; however, in this study, we only used completely flexible and rigid states.

In the third state, we programmed the flexible tail of the robot to periodically stiffen and relax during pivotal moments of the gait cycle. We investigated two distinct gait-stiffening sequences for the purpose of our study Supplementary Video/0:53-1:08 s).

**FIGURE 4:**
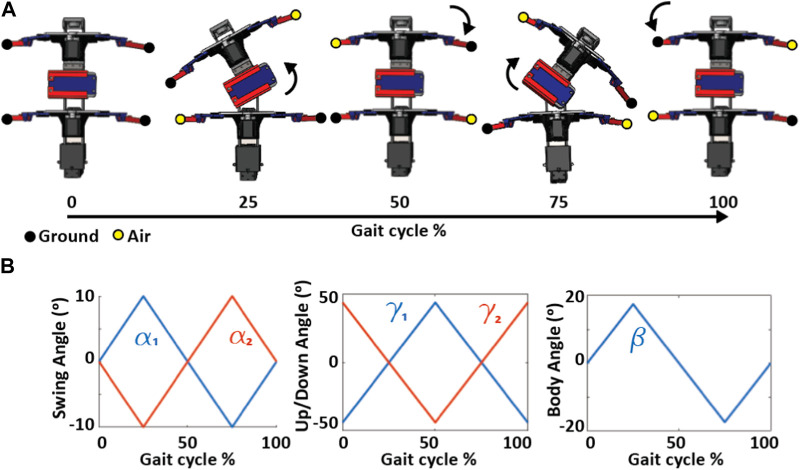
Joint angles and leg contact phases of a diagonal robot gait. **(A)** Body configuration and contact phases of the legs (yellow, in the air; black, on the ground) during the gait cycle. **(B)** Swing (*α*) and up/down(*γ*) angles of legs in front and back segments (*α*_1_, *γ*_1_ and *α*_2_, *γ*_2_) and body angle (*β*) as a function of the gait cycle.

**FIGURE 5:**
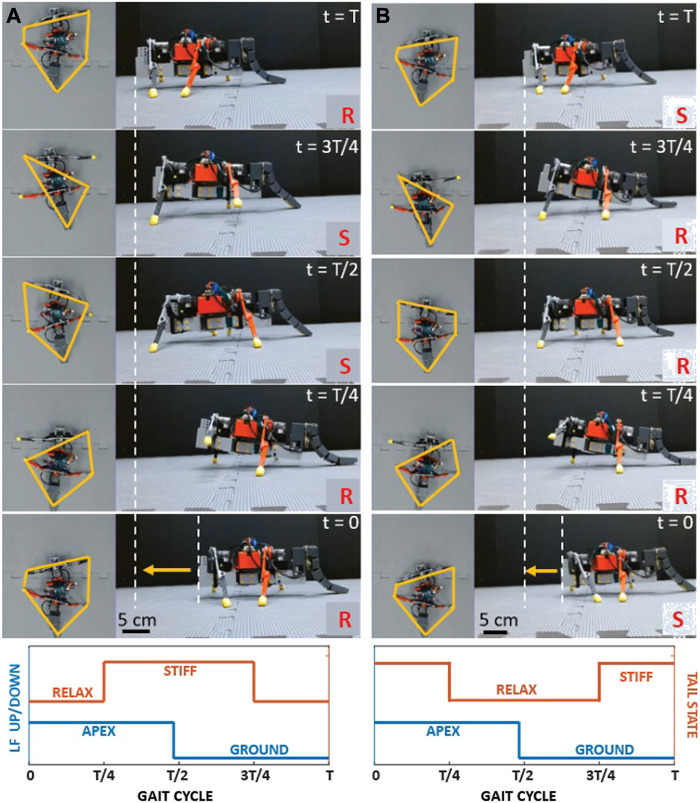
Walking with two different tail actuation sequences. Snapshots from the experiments at t = [0, T/4, T/2, 3T/4, T] s where T is the cycle length. The yellow polygon in the top view images shows the support polygon (the polygon that connects the legs to the ground and the tail). Yellow arrows at t = 0 show the displacement of the robot after a cycle. The letters S and R in the images represent the stiff and rigid state of the tail, respectively. The graphs at the bottom row show the tail state with respect to the up/down state of the left front (LF) leg. **(A)** Tail sequence A, which was consistently used throughout the paper. **(B)** Tail sequence 2 (Supplementary Video/0:53-1:03 s).

In the first sequence, tail stiffening occurred at 50% of the gait cycle (Figure 4), the midpoint of the swing phase of the front left leg, i.e., when it was reaching forward. Tail relaxation occurred at 100% of the gait cycle at the same instant during the swing phase of the front right leg. The stiff tail provided additional support to the robot, supplementing stability in conjunction with the front right leg and the back left leg during the flight phase. When the tail relaxed, all four legs of the robot were in contact with the ground, ensuring continuous stability. The stiff tail not only provided a counterbalance but also played a significant role in propelling the robot forward. On a smooth, flat surface, the robot with this configuration maintained a straight trajectory and covered an optimal distance in a cycle (Figure 5A).

In contrast, the second sequence presented a different outcome. In this sequence, the tail stiffened just before the front right leg touched the ground, with the body curved to the left. The tail was relaxed when the front left, and back right legs were in the air, leading to an unstable stance as the robot had to rely solely on the front right leg and the left back leg without any assistance from the tail. This imbalance led to the robot tilting backward. This configuration consistently deviated from a straight trajectory on a smooth, flat surface, veering to the right, indicative of a foot drop sequence. This gait-stiffening sequence resulted in covering less distance per cycle compared to the first sequence (Figure 5B).

Given these observations, we continued our experiments using the first sequence. It offered a clear advantage in stability and locomotion, making it a more efficient model for our robotic design. However, this does not rule out the potential that other gait-stiffening sequences might bring to the table. For future work, a more comprehensive study could explore other gait-stiffening sequences and their respective impacts on locomotion.

## 3 Experimental results

The effectiveness of the controlled flexible tail on the robot’s locomotion was tested and compared to identical tests carried out on the robot with a rigid tail and the robot with no tail. The experiments aimed to simulate diverse and challenging terrains that the robot might encounter in real-world scenarios. Four scenarios were chosen to test the robot’s locomotion capabilities: (1) walking on a flat surface (indoor), (2) climbing a series of steps (indoor), (3) walking on inclined surfaces (indoor), and (4) traversal of rough, natural terrain (outdoor). For each indoor experiment, there were five trials completed. The robot began at the same location for each trial, and the body position was kept constant by returning the servo motors to their starting position before each test was conducted. In all of the experiments, the robot walked with a diagonal gait in which the diagonal legs are on the ground at 50% of the stance phase while undulating its body, (Ozkan-Aydin et al., 2020; Ozkan-Aydin and Goldman, 2021). The experiments were captured using a side view and a vertical aerial view using two Logitech C920 pro webcams.

To investigate the effectiveness of the flexible tail in facilitating stable and adaptive locomotion in natural environments, a series of outdoor experiments were conducted on diverse terrains, including mulch, pebbles, and sand. The experiments were designed to demonstrate the challenging and unpredictable characteristics of natural environments and to evaluate the robot’s ability to maintain balance and stability on such surfaces.

### 3.1 Flat terrain

The primary objective of this experiments was to evaluate the robot’s capacity to maintain a straight trajectory while navigating flat, smooth terrain, which is an essential requirement for various practical applications in the real world (Supplementary Video/1:09-1:42 s). The robot’s velocity was also analyzed for different tail configurations (no tail, rigid tail, and flexible tail), measured in terms of the distance traveled per gait cycle, normalized to the robot’s body length (BL/cycle). The experiments aimed to assess the impact of the tail design and properties on the robot’s locomotion performance, with particular attention to stability, speed, and accuracy.

In the experiments, a black tape was placed on the surface perpendicular to the “starting line”, to qualitatively examine the robot’s stability by observing the deviations from the path in each configuration. Aspects such as foot clearance and rocking were examined to determine their effect on the robot’s balance. Balance disturbances lead to reduced locomotion speed, disruption to the gait cycle, and unexpected turning.

#### 3.1.1 Locomotion with no tail

This experiment was carried out as described in section 3.1. In each of the five trials conducted, the robot could not meet the goal of traveling along a straight line while on level ground. The robot deviated 2.4° ± 0.71° per cycle from the straight path and traveled with a speed of 0.45 ± 0.03 BL/cycle (or 3.75 ± 0.25 cm/s), Figure 7B.

**FIGURE 6:**
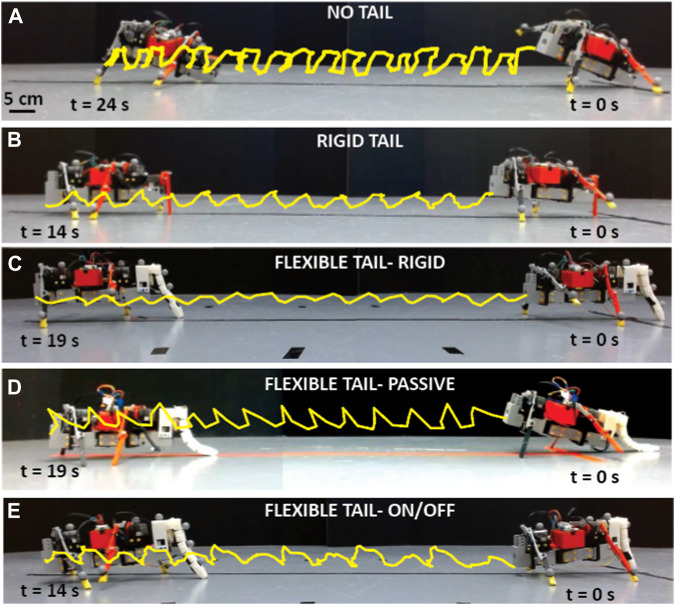
Locomotion on flat terrain with different tail configurations. Experimental snapshots of the robot from a side view with **(A)** no tail **(B)**. A rigid tail **(C)**. A flexible tail with a stiff setting, **(D)**. The flexible tail with a relaxed setting, **(E)** the flexible tail with an alternating relax/stiff setting. The yellow trajectories show the tip trajectory of the robots during walking (Supplementary Video/1:09-1:42 s).

**FIGURE 7:**
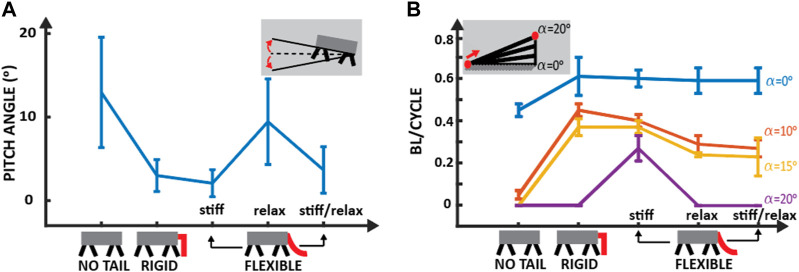
Locomotion on a smooth surface with no tail, rigid tail, and flexible tail. **(A)** Mean and standard deviation of the pitch angle, the angle between the robot’s longitudinal axis and the horizontal plane (see subfigure), for different tail configurations on a flat surface. **(B)** The mean and standard deviation of body length (BL) per cycle for locomotion on smooth flat (blue), 10^*o*^ (red), 15^*o*^ (yellow), and 20^*o*^ (purple) terrain with different tail configurations. Each experiment was repeated at least five times with a minimum of four cycles per run.

This test revealed many problems in the robot’s locomotion without a tail. Firstly, the robot’s body struck the ground twice during each gait cycle. The front of the robot pitched upwards (12.95° ± 6.6°) from the horizontal axis during the swing phase, causing the rear of the robot to strike the ground before foot placement could occur (Figure 6A; Figure 7A). Secondly, the rear legs of the robot did not have sufficient ground clearance during the swing phase, resulting in the rear swinging leg dragging along the ground. Finally, the front legs were found to have excessive ground clearance, leading to increased motion perpendicular to the ground, rather than the desired motion parallel to the ground. These three problems were inexplicably linked to the upward pitching of the robot and caused the robot’s diagonal gait to be asymmetrical and uneven. This impaired the robot’s balance, in turn hindering the robot’s ability to travel along a straight line.

#### 3.1.2 Locomotion with rigid tail

Five replications of the previous experiment were conducted with identical protocols (Figure 6B). Quantitative analysis showed a substantial enhancement in locomotion speed (0.61 ± 0.09 BL/cycle or 5.1 ± 0.75 cm/s), a 34% increase compared to locomotion with no tail (Figure 7B). However, the robot still could not travel in a straight line and deviated from the path 3.13° ± 1.72° per cycle with a rigid tail.

The tail successfully raised the robot’s rear, eliminating any contact of the body with the ground. Upon close examination of the experiments, it was found that there was still an unpredictable and unwanted variance from the gait cycle. This occurred during the swinging phase of the front right leg. The front right leg made contact with the ground before the back left leg, interrupting the diagonal gait of the robot. This caused the front of the robot to pitch with 3.0° ± 1.91° and the tail to lift off the ground. There was further instability when the tail re-made contact with the ground.

#### 3.1.3 Locomotion with flexible tail

Before the experiments were run on the flexible tail, the different tail states/configurations were evaluated. The tail could be configured in the following three ways: **(1)** a relaxed flexible tail where there was no tension being applied (Figure 3B-left), **(2)** a rigid tail where the servo motor was applying constant tension (Figure 3B-right), or **(3)** an alternating flexible to rigid tail, which the rotation of the servo motor would control.

It was found that the flexible tail with constant rigidity provided the best stability (less vertical oscillation, 2.08° ± 1.61°) to the robot while walking on flat, smooth terrain (Figure 6C) compared to other tail configurations (no tail = 12.95° ± 6.6°, rigid tail = 3.0° ± 1.91°, flexible tail-passive = 9.44° ± 5.13° and flexible tail-On/off = 3.67° ± 2.78°). A further improvement in the robot’s ability to travel in a straight line was observed in this test. The robot was capable of traveling along a straight path with 0.71° ± 0.98° rotation/cycle and 0.60 ± 0.04 BL/cycle (or 5 ± 0.33 cm/s) forward displacement, whereas the other tension settings, relaxed and alternating flexible/rigid tail, resulted in 1.48° ± 1.77° and 2.5° ± 1.77° rotations/cycle, respectively. The robot’s movements were smoother and more consistent due to the lack of sudden lurches or pitching. It was found that the side-to-side undulations of the tail, although passive and slight, helped maintain ground contact throughout all stages of the gait cycle. The front leg was still in contact with the ground just before the opposite back leg. However, this no longer caused instability. This is because the ground contact of the tail still provided sufficient support to stop rapid pitching.

Despite being in a rigid state, residual flexibility in the tail emerged as a crucial element in augmenting the robot’s stability and locomotion. This allowed the tail to make slight adjustments automatically when traversing the terrain. When foot strike occurred, the tail moved towards the body momentarily. During the flight phase, the tail quickly moved in the opposite direction away from the body until the tension of the reel limited it. The subtle movements of the tail played a pivotal role in ensuring that the tail remained in contact with the ground, preserving the overall stability of the robot with seamless transitions between gait phases. The robot’s stance position was now altered; the front of the robot was rotated slightly downwards. During the swing phase, the robot pitched marginally upwards until the body angle was approximately parallel to the ground. Then, when the foot strike occurred, the front of the robot pitched downwards to return to the stance position. The swing phase was then again initiated, and the cycle repeated. This resulted in a smooth, consistent gait without sudden and rapid pitching, which would cause the robot to change direction.

The constant stability experienced by the robot led to a further increase in locomotion speed. It traveled at a rate of 0.60 ± 0.04 BL/cycle, similar to the locomotion with the rigid tail.

### 3.2 Inclined surface

In these experiments, we assess the robot’s ability to navigate on an inclined surface with each tail configuration (Supplementary Video/1:43-3:15 s). The experiments involved placing a cardboard runway (50 cm × 85 cm) at various inclinations, ranging from 10 to 20°, and testing the robot’s ability to traverse the entire runway length successfully. Five trials were performed for each incline, and the robot was considered capable of locomotion on an incline if it could make it to the end of the runway successfully (Figure 7B).

#### 3.2.1 Locomotion with no tail

With no tail, the robot could not travel along any incline successfully (Supplementary Video/1:43-1:52 s). It failed to make it to the end of the runway angled at 10° to the horizontal in five consecutive trials. During each of the trials, pitching occurred, resulting in the body striking the ground as well as low rear leg clearance. The cause of this rocking is identical to what is described in Section 3.1.1.

In the flat terrain tests, the pitching of the robot had only a minor effect on its locomotion capabilities. However, when traveling on an inclined surface, the instability caused by the pitching led to the constant slipping of the robot’s rear feet. This made the robot’s travel speed extremely slow (0.05 ± 0.02 BL/cycle). It also resulted in the robot eventually rotating 90° and walking off the runway, failing to meet the success criteria of the experiment.

#### 3.2.2 Locomotion with rigid tail

The addition of a rigid tail allowed the robot to travel along a runway of inclines 10 and 15° with 0.45 ± 0.03 and 0.37 ± 0.04 BL/cycle, respectively (Supplementary Video/1:53-2:08 s). Notably, the robot experienced some degree of slippage during locomotion along these inclines. However, the robot’s hind legs exhibited adequate ground clearance, facilitating forward movement. When the angle was increased to 20°, the slippage of the feet was greater than the distance stepped forward; therefore, the robot traveled in the reverse direction.

#### 3.2.3 Locomotion with flexible tail

The results obtained from our previous experiments have indicated that implementing a flexible tail with constant high tension (Figure 3D) yields the most optimal performance on flat terrain. We, therefore, first conducted our inclined experiments utilizing this tension setting exclusively (Supplementary Video/2:10-2:27 s). As the tail was maintained at a high rigidity throughout the experiment, the robot made continuous contact with the runway during each trial. When tested with this configuration, the robot successfully traveled along a 10, 15, and 20-degree runway with 0.40 ± 0.03, 0.37 ± 0.03, and 0.27 ± 0.06 BL/cycle, respectively. When the incline of the runway was increased to 25°, the robot could not travel along it with any tail type, as it would slide.

For inclines of 10 and 15°, the robot’s motion was only slightly inhibited by some minor slippage of the rear feet. It completed the tests efficiently while traveling along the straight line indicated on the runway. In contrast, the robot experienced more pronounced front and hind legs slippage when traversing inclines of 20°. Despite this, the robot could complete the test at a slightly reduced speed compared to the preceding trials. However, the robot’s performance was severely impaired when the angle of the incline was further increased to 25°. In this case, the robot could not make any net progress up the incline, as it slid back to its previous point after each step. It remained here indefinitely, not moving upwards along the track but also not moving downwards and falling off. This suggests that if a change was made to increase the feet’ grip, the robot could move along this incline successfully.

We also conducted experiments with two different flexible tail settings: a flexible tail in a relaxed setting and a flexible tail in a stiff/relaxed setting. In the relaxed setting (Supplementary Video/2:28-2:52 s), the robot successfully climbed both 10-degree and 15-degree inclines. However, some directional deviations were noted towards the end of the inclines. Specifically, the robot tended to deviate from its straight trajectory, shifting to the left or right. Numerical results indicated a locomotion speed of 0.29 ± 0.04 BL/cycle on a 10-degree incline, decreasing slightly to 0.24 ± 0.01 BL/cycle on a 15-degree incline. When attempting a 20-degree incline, the robot could not successfully climb, continually sliding down and sometimes veering to the sides of the board.

For the stiff/relaxed setting (Supplementary Video/2:55-3:15 s), similar results were obtained. The robot again successfully ascended the 10-degree and 15-degree inclined planes. However, it exhibited a similar pattern of directional deviation near the ends of the slopes, veering left or right after a few cycles. The recorded locomotion speeds were 0.27 ± 0.04 BL/cycle for the 10-degree incline and 0.23 ± 0.09 BL/cycle for the 15-degree incline. As with the relaxed setting, the robot failed to ascend the 20-degree incline, persistently sliding down and occasionally moving sideways.

### 3.3 Stepped terrain

The stepped terrain experiments were split into two sections: (1) upward and (2) downward climbing (Supplementary Video/3:16-4:53 s). As detailed below, both sections followed identical procedures and had the same success criteria. The only difference between the two sections was that in the upward climb test, the robot initiated from the ground in front of the first step and ascended the steps. In contrast, in the downward climb test, the robot commenced from the top step and descended the ‘runway’ towards the ground, where the ground was considered the sixth ‘step.’

The stair setup comprised six steps of uniform height (2.5 cm) and width (25 cm) made of foam boards (Foamular, HomeDepot). The primary objective was to assess the robot’s climbing ability with different tail configurations, with the ultimate goal of climbing as many steps as possible without falling off the runway or failing to make it to the end. The criterion for a successful climb was that all four feet of the robot should make contact with the steps of the stairs. The total number of steps climbed was then counted over five trials for each tail configuration, with a maximum score of 30 possible, and then normalized by the total number of step cycles.

#### 3.3.1 Upward climb with no tail

Without a tail, the robot failed to climb any steps, meaning a result of 0/30, and rotated 30 ± 5^*o*^ per cycle (Supplementary Video/3:16-3:37 s). It was observed during the previous experiments that on flat terrain, the front of the robot pitched up during locomotion, and the rear legs’ ground clearance was extremely small. These two factors impaired the robot while climbing. During each trial, the robot placed its front legs on the first step. It moved forward on that step until the rear legs reached the edge of the step. When this happened, the rear legs could not lift enough to clear the ledge. The body was also making contact with the step. As a result, the robot was stuck indefinitely. The result of this experiment was significant, as it showcased the robot’s inability to climb or navigate over obstacles without getting stuck.

#### 3.3.2 Upward climb with rigid tail

**FIGURE 8:**
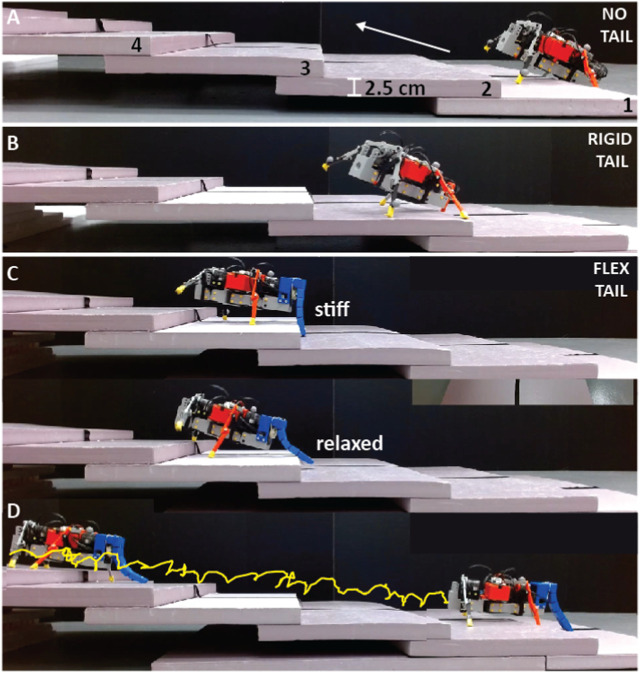
Experimental snapshots of the robot climbing up a series of steps. **(A)** Without a tail (the robot could not climb onto the track), **(B)** With a rigid tail (robot fell from the track at the second step), and **(C, D)** With a flexible tail that is periodically stiffened and relaxed. The stairs were made of 2.5 cm height foam blocks. The yellow trajectory shows the robot’s tip during climbing (Supplementary Video).

**FIGURE 9:**
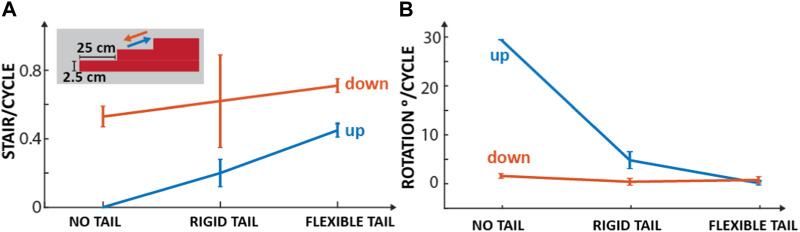
Up/down stair climbing with different tail configurations. **(A)** Mean and standard deviation of stairs climbed up (blue) and down (red) per cycle without a tail, with a rigid tail, and with a flexible tail over five trials. The inset shows the dimensions of the stairs. **(B)** Mean and standard deviation of the rotation of the body per cycle for the same experiments.

There was an improvement in the robot’s climbing ability when a rigid tail was added. The robot successfully climbed 8/30 steps with 0.2 ± 0.08 stairs/cycle over five trials (Figure 9A) with a 5.34 ± 1.75^*o*^ rotation/cycle (Supplementary Video/3:38-4:06 s). With the addition of the rigid tail, the robot could climb onto the first step in every trial. The robot no longer pitched upwards during locomotion, meaning the hind legs had sufficient ground clearance to reach the first step. The robot encountered difficulties moving forward on the second step and attempting to climb onto the next step (Figure 8B). This was due to the lack of contact between the tail and the ground, as the robot’s legs were on a higher elevation surface than the tail, which led to the loss of support and stabilization. Consequently, the robot’s gait reverted to its previous state without a tail, where the front end would incline upwards, and the rear legs would have limited to no ground clearance. When the tail approached the step’s edge, it became stuck, obstructing the robot’s progress. Despite being immobilized, the robot continued moving, but without any forward motion along the track. The front legs extended forward, but the tail constrained the robot in place.

In most trials, the robot continued to move forward until, due to slipping of the feet, it began to rotate to one side and ultimately move off the track. In two instances, sudden jerking forward caused by the robot’s instability allowed the robot’s tail to become free and climb to the next step and continue climbing, although this required numerous gait cycles and a considerable amount of time. It was observed that the robot had already started rotating before being freed, leading to the movement toward the track’s edge. Also, when the robot was attempting to climb while on the track, one of the hind legs made insufficient clearance to climb over the step, resulting in additional rotation and ultimately causing the robot to fall off the track.

It was evident in this test that the length of the robot’s tail is a critical factor in determining its climbing ability. It must be noted that although the robot can climb better with a rigid stick-type leg, this rigid leg can easily get caught on obstacles, not just when climbing. It also does not offer sufficient ground support because the contact area of the leg is small. Increasing the tail’s length increases the clearance height of the robot’s rear and provides more support during climbing, enhancing the robot’s stability and balance. However, longer tails are more likely to get caught on objects or ledges, leading to potential failures during locomotion. Meanwhile, shorter tails decrease the probability of getting stuck but also decrease the support provided during climbing and flat ground traversal, leading to reduced stability and insufficient ground clearance.

#### 3.3.3 Upward climb with flexible tail

Making the tail longer and more flexible allows the robot to adapt the height of its backside to the environment and provide more support during climbing. For this experiment, the tail was configured to stiffen and relax periodically (Supplementary Video/4:07-4:37 s). This tail state is described in Section 2.2.3. In this test, the robot successfully climbed 30 steps out of a possible 30, with 0.45 ± 0.04 stairs/cycle and 0.66° ± 0.41° rotation/cycle, over 5 trials (Figure 9).

When the tail is stiffened, the robot can climb to a higher step by providing support in the form of constant ground contact, resulting in a large clearance distance between the legs and the ground. While the robot is on the higher step, the body remains parallel, resulting in smoother locomotion. However, due to the length of the tail, it can get stuck when it reaches the edge of a step, and the robot ceases to move forward.

To prevent this, the tail relaxes by releasing the tension on the cables. This allows the tail to move over the step and for the robot to continue climbing. Due to the periodic nature of the stiffening and relaxation of the tail, the tail may inhibit movement in some cases, and the robot may appear stuck momentarily. This occurs when the tail meets a step in its stiffened state. The robot then attempts to move forward for a brief period. During the forward swing, the tail is relaxed, and the robot accelerates rapidly forward. The feet of the robot slides along the surface, and as a result, the robot moves a further distance forward than in a typical cycle. The stiffening of the tail also thrusts the robot, pushing it upward and forward at a specific instant during the gait cycle to increase locomotion effectiveness.

#### 3.3.4 Upward climb with flexible tail with sensing

An extra set of trials were conducted on the upward climb experiment, incorporating a touch sensor near the end of the tail to control the state of the tail (Supplementary Video/4:38-4:53 s). The robot climbed 26 out of 30 steps with 0.32 ± 0.04 stairs/cycle and 1.92° ± 1.25° rotation/cycle. During the tests, the tail remained rigid until it came into contact with a step. When the touch sensor recorded a force, it relaxed the tail until it was no longer in contact with the step. The tail then returned to its rigid state and continued traveling forward.

Using a sensor allowed the tail to be stiffened when attempting to climb, providing important stability and foot clearance to climb upwards successfully. It also allowed the tail to relax when encountering obstructions and prevented the robot from getting stuck. Although this tail configuration significantly improved the robot’s climbing ability compared to a completely rigid tail, it performed worse than the open-loop flexible tail. This was because the tail relaxation and stiffening timing were altered so that it was not occurring at the optimal stages during the gait cycle (Section 2.2.3). Additionally, our reliance on a single sensor located on the tail meant that the sensor could only detect stairs when the tail made contact from a specific point, resulting in occasional misses. To address this issue in the future, implementing a sensor array across the entire tail could provide more accurate data and control.

#### 3.3.5 Downward climb with no tail

When the robot has no tail attached, it performs better at climbing downwards than upwards. The robot successfully traveled down 15 steps out of a possible 30 with 0.53 ± 0.06 stairs/cycle and 2.12 ± 0.47^*o*^ rotation/cycle over six trials. Previous experiments showed that when traveling along flat terrain, the robot pitches upwards, causing the rear to make contact with the ground. This still occurred when climbing downwards, but when the robot moved to a lower step, it violently pitched downwards, causing the front to hit the ground or runway. This caused further disturbance to the robot’s gait, leading to instability and inconsistencies in the robot’s direction of travel.

#### 3.3.6 Downward climb with rigid tail

The robot successfully traveled down 21 steps with 0.62 ± 0.27 stairs/cycle and 0.92 ± 0.71^*o*^ rotation/cycle when a rigid tail was attached. The robot’s locomotion patterns were made more consistent by adding a tail. However, upon each foot placement, the robot pitched downwards, and the tail briefly lost contact with the supporting surface. This caused problems when moving onto a lower step. In those cases, the robot pitched to an even more extreme angle, resulting in the front of the robot making contact with the surface. Similar to the previous experiment, these collisions with the runway resulted in negative consequences such as misdirection and less efficient locomotion, albeit to a lesser degree.

#### 3.3.7 Downward climb with flexible tail

With the addition of the flexible tail, the robot climbed down 30 out of a possible 30 steps with 0.71 ± 0.04 stairs/cycle and 1.3 ± 0.7 rotation/cycle. Different from the upward climbing, where the tail was periodically stiffened and relaxed, in these experiments, the tail was configured so that it was fully flexible, i.e., there was no tension in the cables and no periodic motion of the reel. This configuration of the tail made the robot more adaptable to its terrain. When stepping off each step, the tail extends to the point where it is almost entirely vertical. At this instant, the bottom of the tail is in direct contact with the ground (the narrow end piece of the tail). Simultaneously, the front leg springs bend, and the front of the robot makes contact with the ground. However, as the tail still supports the robot, there is little impact felt by it, and there are no deviations from the path. At all other stages, the tail is flexed away from the robot’s body. This allows the tail to continue to offer support when the legs of the robot have moved to a lower step.

### 3.4 Outside experiments

To demonstrate the tail function on locomotion, outside experiments were carried out on the following terrains: a pebbled surface, mulch ground with plant litter (twigs, leaves, etc.), and sand. The key parameter to be analyzed in this experiment was how the robot reacted to uneven terrains and whether adding a flexible tail improved these interactions. This was done qualitatively. In this section, tests were only performed on the robot with no tail and with a flexible tail with stiff/relaxed configuration.

#### 3.4.1 Mulch terrain

**FIGURE 10:**
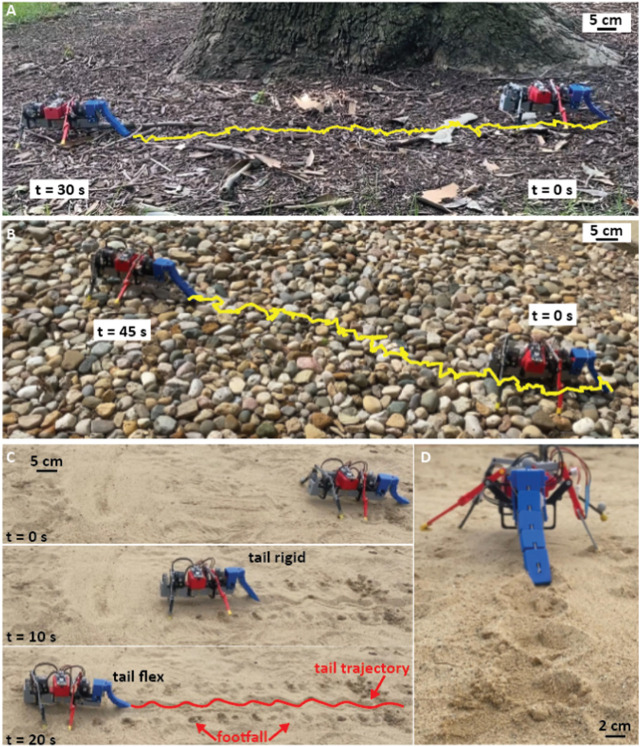
Outside demonstrations. **(A)** Side view of the robot while walking on mulch surface. Yellow trajectory shows the trajectory of the tip of the tail during walking (t = 0–30 s) with a speed 4.03±0.15 cm/s, **(B)** Locomotion on a pebbled surface with a speed 2.04±0.6 cm/s. **(C)** Side view of the robot while walking on sand with a speed of 3.42±0.24 cm/s. At t = 0 and 20 s, the tail of the robot is in its relaxed state, and at t = 10 s, it is in its rigid state. The red trajectory shows the tail trajectory. Also, the footfalls of the robot are highlighted with red arrows, **(D)** Back view of the robot and its tail trajectory while walking on the sand (Supplementary Video-5:37-6:10 s).

Without a tail, the robot could traverse on mulch without being significantly affected by the softness of the surface (Figure 10A) compared to its locomotion on a smooth flat surface. However, when the robot encountered organic debris like leaves and fallen twigs, the legs of the robot would drag the debris along, reducing the robot’s velocity (Supplementary Video/5:23-5:34 s). When a flexible tail was added, which exhibited periodic stiffening and relaxation, the robot was more resilient to these obstacles and locomoted at 4.03 ± 0.15 cm/s (Supplementary Video/4:54-5:06 s). The tail’s flexibility allowed it to absorb the impacts of the obstacles and facilitate the robot’s smooth movement.

#### 3.4.2 Pebbled terrain

With no tail, the robot traveled extremely slowly (∼ zero speed) over pebbled surfaces. The robot’s legs repeatedly got stuck in gaps between stones due to the legs’ low ground clearance and the body’s pitching. This was compounded by the robot’s eventual entrapment, which resulted from one of its legs becoming firmly lodged between stones, rendering it immobile.

When the robot was tested with a flexible tail that exhibited periodic stiffening and relaxation, the locomotion velocity was increased to 2.04 ± 0.6 cm/s (Supplementary Video/5:07-5:21 s). The legs no longer got stuck due to the movement of the tail and the support it provided. Although the tail occasionally became stuck, the periodic relaxation of the tail allowed it to become dislodged quickly without impeding the robot’s overall performance (Figure 10B).

#### 3.4.3 Sand

When traveling on sand, the robot’s rear legs did not lift off the surface as expected but instead were dragged along through the sand. This unexpected movement of the robot’s legs disrupted the gait cycle and subjected the rear legs to increased force and twisting, which the robot was not designed to handle (Supplementary Video/5:37-6:10 s). Continued use of the robot on sand would likely lead to fracturing of the legs or body, as well as damage to the rear motor due to the excessive forces being exerted.

In contrast to the issues encountered when the robot traversed a sandy terrain without a tail, adding a flexible tail proved to be an effective solution (Figure 10C). The tail’s ability to provide constant support and its side-to-side passive undulation was clearly visible in the tracks left by the robot (Figures 10C, D). Specifically, the tail enabled the robot to traverse small divots and uneven surfaces with a velocity of 3.45 ± 0.24 cm/s, which it previously could not navigate effectively (Supplementary Video/5:37-6:10 s).

## 4 Conclusion and future works

This paper describes the development and implementation of a multi-segment, flexible tail with variable rigidity controlled by a cable-driven mechanism. The performance of a sprawling quadruped robot was shown to be significantly improved by conducting experiments that compared the robot’s locomotion with different tail configurations across several indoor and outdoor environments.

The constant ground support provided by the flexible tail is key in maintaining stable locomotion. This ensures a predictable gait cycle that stops unexpected turning and slipping, as experienced by the robot in the other two tail configurations. This leads to an increase in the locomotion speed and efficiency of the robot. Moreover, the constant ground support also enables the robot to travel along an inclined surface with an angle of up to 20° to the horizontal.

The variable stiffness of the tail plays a significant role in enhancing the robot’s climbing ability. The versatility and adaptability of the tail allows the robot to successfully overcome obstacles in its path when climbing without the need for external sensing.

All of the improvements mentioned above to the robot’s locomotion were observed when testing on natural terrain occurred. The adaptability of the flexible tail greatly improved the robot’s locomotion capabilities when traveling on pebbled, mulch, and sandy terrain.

The idea of implementing a rigid curved tail instead of a variable stiffness tail presents an interesting alternative approach. A rigid curved tail might offer similar benefits in terms of ground support and stability during locomotion on flat surfaces. Moreover, its curved structure could potentially allow it to slide over obstacles, such as stairs, thereby aiding in climbing tasks. However, a rigid curved tail might not provide the same level of adaptability that the variable stiffness tail offers. In scenarios where the robot encounters uneven or outdoor terrains with uncertain and varying obstacles, such as pebbles, mulch, or sand, the lack of knowledge regarding the size and shape of the obstacles or the roughness of the surface poses challenges. The variable stiffness tail’s capability to adjust its shape based on environmental cues becomes advantageous in terms of maintaining stability and maneuverability. In contrast, a rigid curved tail might become hindered or fail to provide adequate support, ultimately restricting the robot’s ability to navigate through these intricate environments successfully.

The choice between a rigid curved tail and a variable stiffness tail warrants careful consideration, as it involves balancing adaptability, stability, and locomotive performance in diverse and complex scenarios. Future studies and analyses will be necessary to assess the optimal tail design concerning specific environmental conditions and locomotion requirements for soft robotic systems.

A limitation of the open-loop system employed in conjunction with the flexible tail is its inability to change the state of the flexible tail automatically. The configuring of the tail, i.e., full flexibility, constant rigidity, or periodic rigidity, must occur before the experiments begin, based on the terrain. However, future work will focus on integrating various sensors to automatically change the tail’s state according to the terrain properties. This will allow the robot to adapt to its terrain and change its gait to effectively navigate obstacles, with the goal of applying the robot autonomously in outdoor, real-world environments. Our testing of the incorporation of a touch sensor to the end of the tail (Section 3.3.4), has shown promising results in aiding the robot during stair climbing, with the relaxing and stiffening of the tail determined by the sensor (Supplementary video).

With the implementation of a closed-loop system, the robot would travel with the tail in its rigid state when the surface is smooth or flat. If an obstacle is sensed in front of the robot, the tail would periodically relax and stiffen to prevent entrapment. Similarly, if the surface underneath the robot were to fall off, the tail would fully relax, providing support while the robot pitched downwards. These advances may lead to the autonomous application of the legged robot in outdoor, real-world environments, where it can be used for various tasks, such as search and rescue, environmental monitoring, and inspection.

## Data Availability

The original contributions presented in the study are included in the article/Supplementary Material, further inquiries can be directed to the corresponding author.
